# Stevioside Prevents Wear Particle-Induced Osteolysis by Inhibiting Osteoclastogenesis and Inflammatory Response via the Suppression of TAK1 Activation

**DOI:** 10.3389/fphar.2018.01053

**Published:** 2018-09-26

**Authors:** Jiahong Meng, Chenhe Zhou, Bin Hu, Mengmeng Luo, Yute Yang, Yangxin Wang, Wei Wang, Guangyao Jiang, Jianqiao Hong, Sihao Li, Haobo Wu, Shigui Yan, Weiqi Yan

**Affiliations:** ^1^Department of Orthopedic Surgery, The Second Affiliated Hospital, Zhejiang University School of Medicine, Hangzhou, China; ^2^Orthopedic Research Institute of Zhejiang University, Hangzhou, China; ^3^Department of Plastic Surgery, The First Affiliated Hospital, Zhejiang University School of Medicine, Hangzhou, China

**Keywords:** aseptic loosening, osteoclast, NF-κB – nuclear factor-kappa B, MAPK, TAK1

## Abstract

Aseptic loosening and periprosthetic osteolysis are the leading causes of total joint arthroplasty failure, which occurs as a result of chronic inflammatory response and enhanced osteoclast activity. Here we showed that stevioside, a natural compound isolated from *Stevia rebaudiana*, exhibited preventative effects on titanium particle-induced osteolysis in a mouse calvarial model. Further histological assessment and real-time PCR analysis indicated that stevioside prevented titanium particle-induced osteolysis by inhibiting osteoclast formation and inflammatory cytokine expression *in vivo*. *In vitro*, we found that stevioside could suppress RANKL-induced osteoclastogenesis and titanium particle-induced inflammatory response in a dose-dependent manner. Mechanistically, stevioside achieved these effects by disrupting the phosphorylation of TAK1 and subsequent activation of NF-κB/MAPKs signaling pathways. Collectively, our data suggest that stevioside effectively suppresses osteoclastogenesis and inflammatory response both *in vitro* and *in vivo*, and it might be a potential therapy for particle-induced osteolysis and other osteolytic diseases.

## Introduction

Total joint arthroplasty (TJA) is considered a successful surgical procedure for end-stage joint diseases, such as osteoarthritis and rheumatoid arthritis. As reported, approximately 1.2 million TJA procedures are performed annually in the United States, and that number is expected to increase to 3.8 million by the year 2030 (Teeny et al., 2003; Parvizi et al., 2017). Although progress has been made in the efficacy of TJA, periprosthetic osteolysis and subsequent aseptic loosening continue to be one of the leading causes of arthroplasty failure (Bozic et al., 2009). Production of titanium (Ti), ultra-high molecular weight polyethylene (UHMWPE), or cement wear debris following TJA is deemed to play a critical role in the process of osteolysis (Noordin and Masri, 2012). Considerable studies have shown that chronic inflammatory response and increased osteoclast-related bone resorption, which occur in response to implant-derived wear debris, might be responsible for osteolysis (Masui et al., 2005; Holding et al., 2006; Abu-Amer et al., 2007; Nam et al., 2017).

Generally, wear debris generated from the prosthetic joint articular surface causes the recruitment of cells, including macrophages, fibroblasts, lymphocytes, and osteoclasts. These cells, especially macrophages, are stimulated to secrete proinflammatory cytokines, such as tumor necrosis factor (TNF)-α, interleukin (IL)-1β, IL-6, IL-11, nitric oxide (NO), and prostaglandin E2 (PGE2), into periprosthetic tissues, exacerbating the inflammatory response. These cytokines impair osteoblast activity and cause the overexpression of receptor activator of nuclear factor-κB ligand (RANKL) in osteoblasts (Pioletti and Kottelat, 2004; Lee et al., 2012). RANKL mediates osteoclast differentiation and function via activating a series of signaling cascades, such as nuclear factor-kappa B (NF-κB) and mitogen-activated protein kinase (MAPK) signaling pathways (Feng, 2005). The elevated RANKL expression induces excessive osteoclast formation and bone resorption, which ultimately result in periprosthetic osteolysis (Purdue et al., 2007). Therefore, drugs that suppress inflammatory response and/or RANKL-induced signaling pathways have great potential to prevent wear particle-induced osteolysis and other osteolytic diseases.

Stevioside, a diterpene glycoside isolated from *Stevia rebaudiana* (commonly known as sugar leaf), has shown a wide range of pharmacological effects, including anti-inflammatory (Yingkun et al., 2013), immunomodulatory (Boonkaewwan and Burodom, 2013), anti-diabetic (Wang et al., 2012), and cardioprotective (Ragone et al., 2017) properties. Researchers have shown that stevioside can inhibit pro-inflammatory cytokine secretion from LPS-induced rat peripheral blood mononuclear cells (Noosud et al., 2017). In addition, stevioside has been demonstrated to suppress the release of inflammatory cytokines by interfering with NF-κB and MAPK signaling pathways in LPS-stimulated RAW264.7 cells (Fengyang et al., 2012). However, little is known about the effects of stevioside on osteoclasts and osteolytic diseases. Given the importance of inflammatory cascades and NF-κB/MAPK signaling pathways in the process of osteoclast-related osteolysis, as well as the suppressive effect of stevioside on LPS-induced inflammatory response through these pathways, we hypothesized that stevioside might be a novel candidate for treatment of particle-induce osteolysis by inhibiting osteoclastogenesis and inflammatory response.

In this study, we showed that stevioside prevented particle-induced osteolysis by inhibiting osteoclast formation and inflammatory cytokine expression *in vivo*. Further *in vitro* study confirmed that stevioside attenuated RANKL-induced osteoclastogenesis and Ti particle-induced inflammatory response by disrupting the phosphorylation of TGF-β-activated kinase 1 (TAK1) and subsequent activation of NF-κB/MAPK signaling pathways.

## Results

### Administration of Stevioside Prevents Ti Particle-Induced Osteolysis in a Mouse Calvarial Model

**FIGURE 1:**
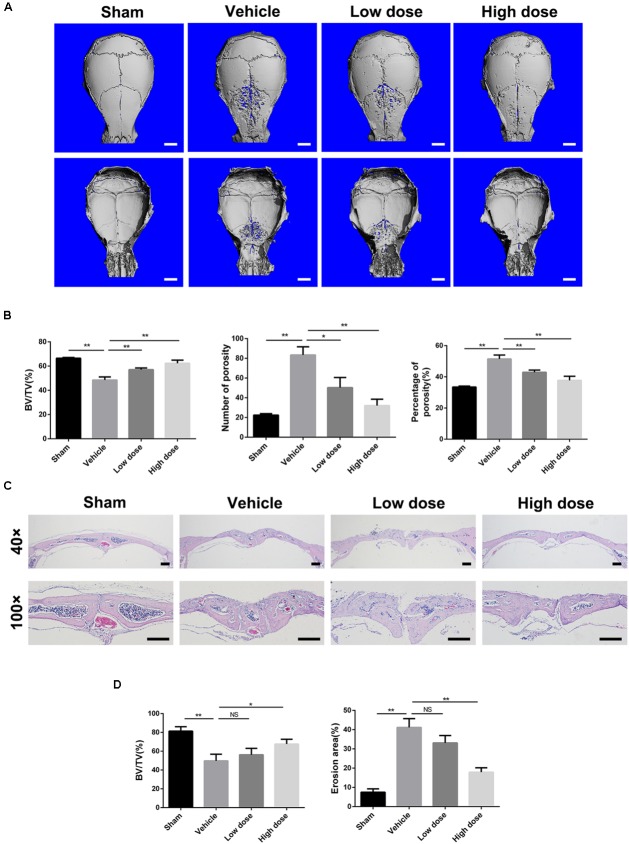
Stevioside prevents Ti particle-induced osteolysis in a mouse calvarial model. **(A)** Representative micro-CT reconstruction images from each group. Scale bars = 1 mm. **(B)** Bone volume against tissue volume (BV/TV, %), number of porosity and the percentage of porosity (%) of each sample. **(C)** Representative H&E staining of calvarial bone sections from sham, vehicle, low and high dose stevioside-treated groups. Scale bars = 200 μm. **(D)** Histomorphometric analysis of BV/TV and erosion area for each sample. Low dose, 10 mg⋅kg^-1^⋅day^-1^; high dose, 30 mg⋅kg^-1^⋅day^-1^. Data are presented as mean ± SD, *n* = 5. ^∗^*P* < 0.05, ^∗∗^*P* < 0.01, NS, not significant, compared with the vehicle group.

To investigate the potential preventative effect of stevioside on pathological osteolysis, we established a Ti particle-induced murine calvarial osteolysis model. Ti particles were embedded under the periosteum at the middle suture of the calvaria in 6-week-old C57BL/6 mice treated without or with stevioside (10 mg⋅kg^-1^⋅day^-1^ and 30 mg⋅kg^-1^⋅day^-1^). After 14 days, calvaria were collected and analyzed by micro-computed tomography (CT) and histology. Micro-CT with 3D reconstruction revealed that mice in the vehicle group suffered from extensive bone erosion on the calvaria compared with the sham group. In contrast, the administration of stevioside attenuated Ti particle-induced osteolysis in a dose-dependent manner (**Figure 1A**). Quantitative analysis of bone parameters presented as bone volume-tissue volume ratio (BV/TV, %), the number of porosity and percentage of porosity, confirmed that the treatment of stevioside significantly reduced the bone loss induced by Ti particles (**Figure 1B**).

Likewise, histological assessment further confirmed the therapeutic effect of stevioside on osteolysis. Hematoxylin and eosin (H&E) staining showed that Ti particles induced severe osteolytic changes in the vehicle group, whereas stevioside treatment effectively prevented osteolysis (**Figure 1C**). Consistent with the micro-CT quantitation, histomorphometric analysis showed that high dose of stevioside significantly reduced the extent of bone erosion induced by the Ti particles, characterized by the reversed quantitative value of BV/TV and erosion area (**Figure 1D**). These data demonstrated that stevioside prevents Ti particle-induced osteolytic bone loss *in vivo*.

### Administration of Stevioside Suppresses Osteoclast Activity and Inflammatory Response *in vivo*

**FIGURE 2:**
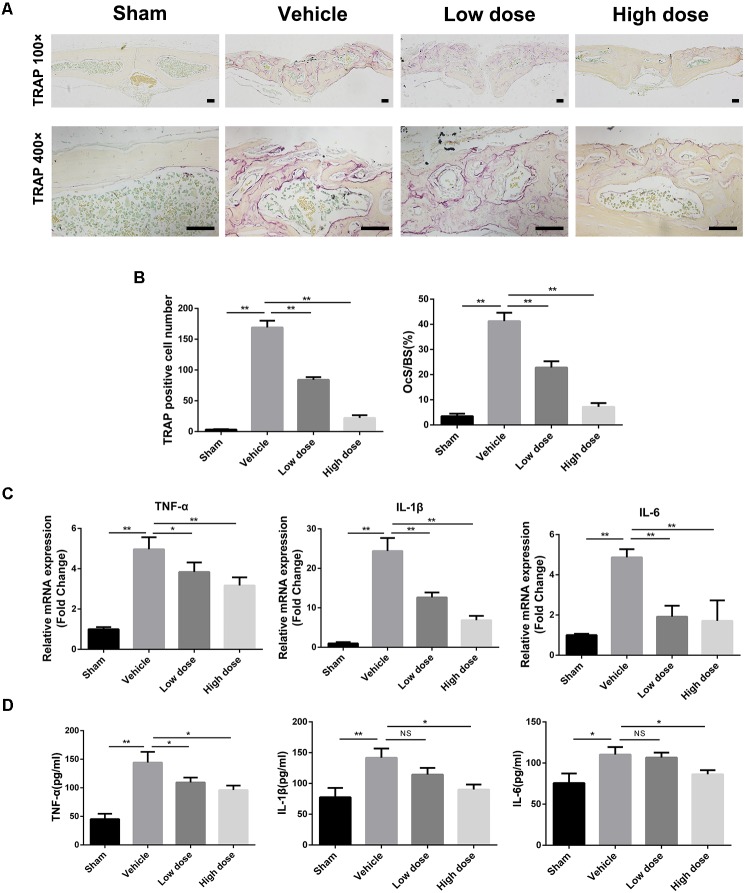
Stevioside prevents Ti particle-induced bone loss by regulating inflammatory response and osteoclast activity. **(A)** Representative TRAP staining of calvarial bone sections from sham, vehicle, low and high dose stevioside-treated groups. Scale bars = 50 μm. **(B)** The number of TRAP-positive osteoclasts, and the percentage of osteoclast surface per bone surface (OcS/BS, %) were assessed. **(C)** The mRNA levels of TNF-α, IL-1β, and IL-6 in calvarial bones were analyzed by real-time PCR. **(D)** The protein levels of TNF-α, IL-1β, and IL-6 in the supernatants of cultured calvaria were measured by ELISA. Low dose, 10 mg⋅kg^-1^⋅day^-1^; high dose, 30 mg⋅kg^-1^⋅day^-1^. Data are presented as mean ± SD, *n* = 5. ^∗^*P* < 0.05, ^∗∗^*P* < 0.01, NS, not significant, compared with the vehicle group.

As chronic inflammatory response and excessive osteoclast activity play a crucial role in wear particle-induced osteolysis, we next investigated the effects of stevioside on them *in vivo*. Tartrate-resistant acid phosphatase (TRAP) staining was performed to detect osteoclast activity. As shown in **Figure 2A**, numerous TRAP-positive osteoclasts accumulated along the eroded bone surface in the vehicle group in comparison with the sham group, while decreased numbers of osteoclasts were observed in the stevioside treatment groups. Histomorphometric analysis revealed that the number of TRAP-positive cells and the percentage of osteoclast surface per bone surface (OcS/BS, %) were shown to be obviously increased in the vehicle group. In contrast, stevioside treatment led to a decrease in both the number of TRAP-positive cells and OcS/BS in a dose-dependent manner (**Figure 2B**).

We next investigate the effect of stevioside on the expression of inflammatory genes by real-time (RT)-PCR analysis. We found increased *TNF-α*, *IL-1β*, and *IL-6* expression in the parietal bones of the vehicle group compared with that in the sham group (**Figure 2C**). However, the expression levels of *TNF-α*, *IL-1β*, and *IL-6* were significantly inhibited by stevioside treatment. Meanwhile, the protein levels of TNF-α, IL-1β, and IL-6 in the medium of cultured calvaria were also examined by ELISA (**Figure 2D**). As expected, the increased protein levels of TNF-α, IL-1β, and IL-6 induced by Ti particles were markedly suppressed by stevioside, which was consistent with the mRNA expression levels.

Taken together, our results suggested that stevioside exerted a strong preventative effect on Ti particle-induced osteolysis by regulating inflammatory response and osteoclast activity. Therefore, following experiments focused on the role of stevioside in osteoclastogenesis and inflammatory response *in vitro*.

### Stevioside Suppresses RANKL-Induced Osteoclast Formation *in vitro* With Negligible Cytotoxicity

**FIGURE 3:**
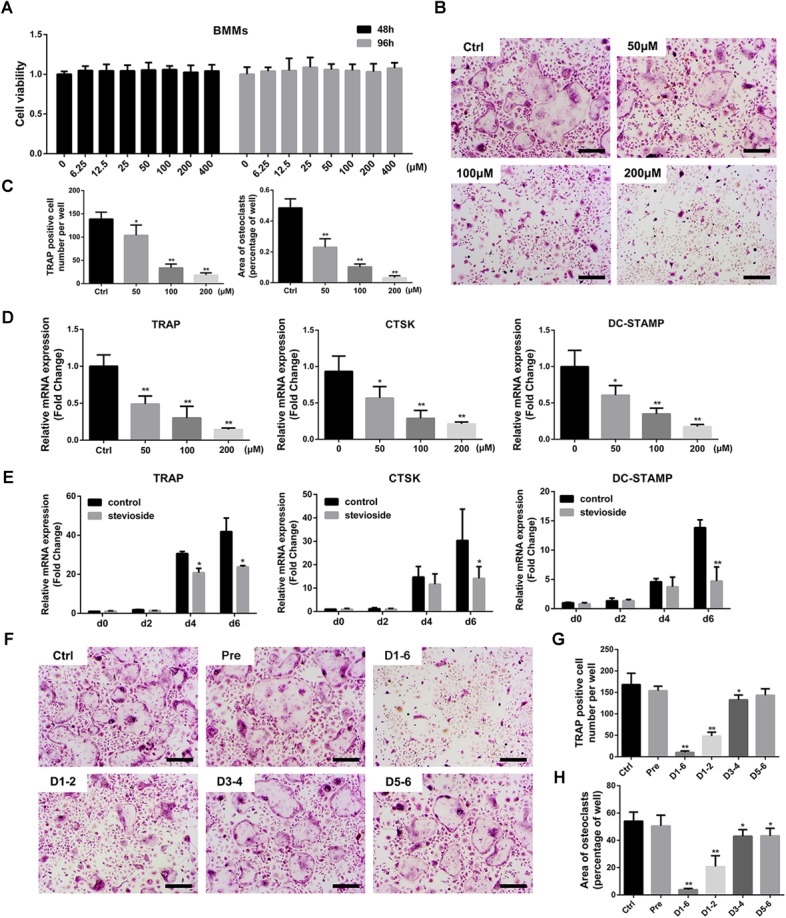
Stevioside inhibits RANKL-induced osteoclastogenesis without cytotoxicity *in vitro*. **(A)** Viability of BMMs exposed to stevioside was measured by CCK-8 assay at 48 and 96 h. **(B)** BMMs were stimulated with M-CSF and RANKL in the presence of the indicated stevioside concentrations for 6 days. Cells were then fixed and stained for TRAP activity. Scale bars = 200 μm. **(C)** The number and area of TRAP-positive cells were analyzed. **(D)** Trap, Ctsk, and Dc-stamp expression in BMMs treated with the indicated stevioside concentrations for 6 days. **(E)** Trap, Ctsk, and Dc-stamp expression in BMMs treated with 200 μM stevioside for 0, 2, 4, and 6 days. **(F)** BMMs were stimulated with M-CSF and RANKL for 6 days, and 200 μM stevioside was added at the indicated days. Cells were fixed and stained for TRAP activity. Scale bars = 200 μm. **(G,H)** The number and area of TRAP-positive cells were analyzed. Data are presented as mean ± SD. ^∗^*P* < 0.05, ^∗∗^*P* < 0.01, compared with the controls.

A cell viability assay was performed to determine the potential cytotoxic effect of stevioside on bone marrow-derived macrophages (BMMs) for 2 or 4 days. Our results showed that stevioside had no cytotoxic effect at concentrations up to 400 μM on BMMs (**Figure 3A**).

To investigate the effect of stevioside on osteoclastogenesis, BMMs were treated with M-CSF, RANKL, and different concentrations of stevioside (0, 50, 100, and 200 μM) for 6 days. A large number of mature TRAP-positive multinucleated osteoclasts formed in the control group (**Figure 3B**). In contrast, the number and area of osteoclasts were significantly decreased by treatment with stevioside in a dose-dependent manner (**Figure 3C**). RT-PCR further confirmed that stevioside treatment inhibited the expression levels of osteoclast-specific genes, including *Trap*, *cathepsin K* (*Ctsk*), and *Dc-stamp*, in a dose-dependent (**Figure 3D**) and time-dependent manner (**Figure 3E**).

To further investigate at which stage of osteoclastogenesis stevioside exerted its inhibitory effect, cells were treated with 200 μM stevioside at day 1–day 2 (early stage), day 3–day 4 (middle stage), day 5–day 6 (late stage), day 1–day 6 (whole stage), and one day before differentiation (pretreatment). As shown in **Figures 3F–H**, the number and size of osteoclasts were dramatically decreased with early-stage administration of stevioside and slightly decreased with middle-stage administration. A small but significant reduction in the area of osteoclasts was observed with late-stage stevioside treatment, but no difference was observed in the number of osteoclasts. Collectively, these data confirmed the suppressive effects of stevioside on osteoclast formation, especially at the early stage of osteoclast differentiation.

### Stevioside Attenuates the Formation of F-Actin Ring and Bone Resorption *in vitro*

**FIGURE 4:**
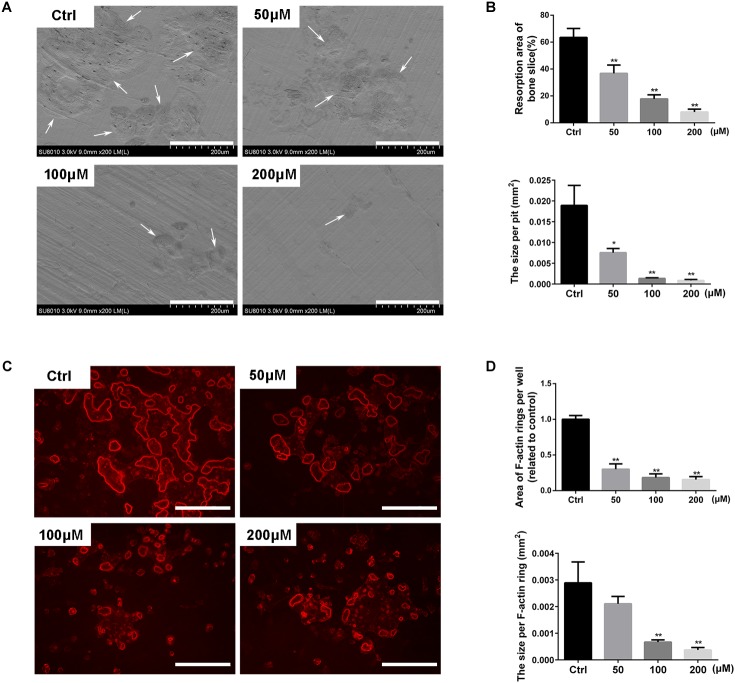
Stevioside inhibits bone resorptive activity of mature osteoclasts. An equal number of BMM-derived mature osteoclasts were seed onto bovine bone slices and treated with indicated concentrations of stevioside for another 2 days. **(A)** Representative scanning electron microscopy (SEM) images of bone resorption pits. Scale bars = 200 μm. **(B)** The resorption area of bone discs was measured using ImageJ. **(C)** Representative fluorescence images of F-actin staining with rhodamine-phalloidin. Scale bars = 200 μm. **(D)** The area of F-actin rings was measured using ImageJ. Data are presented as mean ± SD. ^∗^*P* < 0.05, ^∗∗^*P* < 0.01, compared with the controls.

Since stevioside had shown the effect of inhibiting osteoclast formation, we further detected its effects on osteoclast functions. Mature osteoclasts were plated onto bovine bone slices and treated with different concentrations of stevioside in the presence of osteoclastogenic medium. Bone resorption pits were detected by a scanning electron microscope (SEM). As show in **Figure 4A**, extensively resorbed bone pits were observed in the control group, while a decreased number of resorptive bone pits was observed in the stevioside-treated groups. Bone resorption area and the size per pit were efficiently reduced after the treatment with 50 and 100 μM stevioside, and rare resorption pits were observed on the bone slices treated with 200 μM stevioside (**Figure 4B**).

We also examined the effect of stevioside on F-actin ring formation, which is a discernible and observable characteristic of mature osteoclasts and an essential prerequisite for osteoclastic bone resorption (Akisaka et al., 2001; Feng et al., 2009). Characteristic F-actin ring structures were observed under fluorescence microscopy in the control group, while stevioside partly inhibited the formation of F-actin ring (**Figure 4C**). Statistically, the area and the size of F-actin ring were significantly decreased in the presence of stevioside (**Figure 4D**). Collectively, these findings suggested that stevioside attenuates osteoclastic bone resorption and F-actin ring formation *in vitro*.

### Stevioside Suppresses RANKL-Induced Osteoclastogenesis by Inhibiting the Phosphorylation of TAK1

**FIGURE 5:**
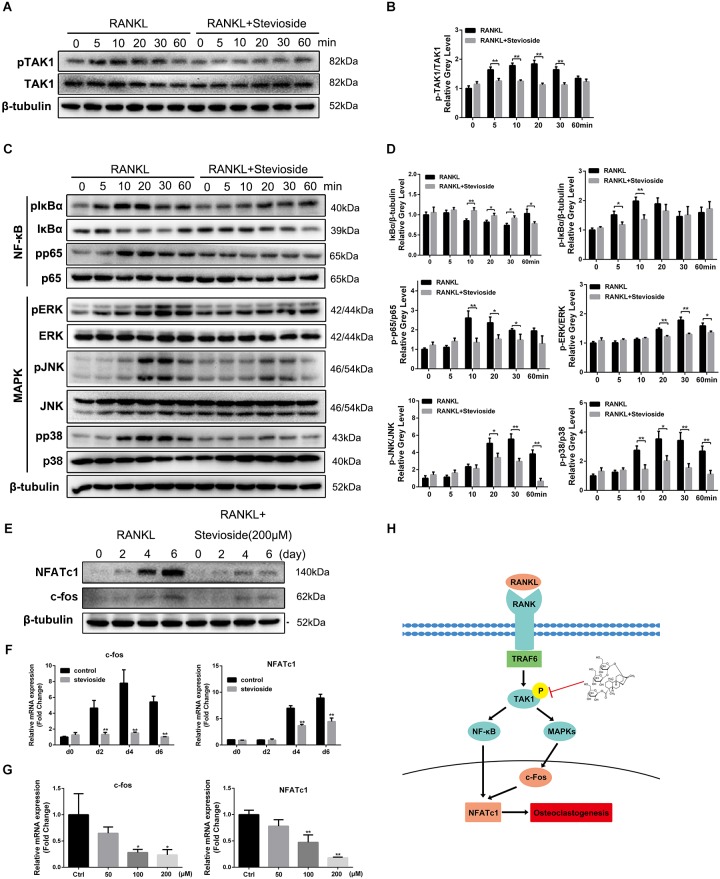
Stevioside inhibits osteoclast differentiation by specifically impairing RANKL-induced phosphorylation of TAK1 and subsequent activation of NF-κB/MAPK signaling pathways. **(A,C)** BMMs were pretreated with or without 200 μM stevioside for 4 h and then treated with 50 ng⋅mL^-1^ RANKL for the indicated periods. Cell lysates were analyzed using western blotting. **(B)** The gray level of phosphorylated TAK1 was normalized relative to total TAK1. **(D)** The gray levels of phosphorylated p65, ERK, JNK, and p38 were quantified and normalized relative to total p65, ERK, JNK, and p38. The gray levels of phosphorylated IκBα and IκBα were normalized to β-tubulin. **(E)** The protein expression levels of NFATc1 and c-Fos in BMMs treated with stevioside for 0, 2, 4, or 6 days. **(F,G)** The mRNA expression levels of NFATc1 and c-Fos in BMMs treated with the indicated stevioside concentrations for 0, 2, 4, or 6 days. RNA expression levels were normalized relative to the expression of GAPDH. **(H)** Schematic representation of the experiments presented in this figure. Data are presented as mean ± SD. ^∗^*P* < 0.05, ^∗∗^*P* < 0.01, compared with RANKL alone.

To illustrate the underlying mechanisms through which stevioside inhibited osteoclast formation and function, we investigated the main signaling pathways involved in osteoclast differentiation. Previous studies revealed that the activation of TAK1 and subsequent signaling pathways including NF-κB and MAPK are the indispensable early signaling event in RANKL-induced osteoclast formation (Feng, 2005; Huang et al., 2006; Yamashita et al., 2007). As shown in **Figure 5A**, the phosphorylation of TAK1 was observed 5 min after RANKL stimulation and peaked at 20 min. However, in the presence of 200 μM stevioside, RANKL-induced TAK1 phosphorylation was significantly decreased. Quantitative analysis also confirmed our observations (**Figure 5B**). In addition, our data showed that the phosphorylation of IκBα and p65 peaked at 10 min after RANKL stimulation, while the phosphorylation levels were suppressed by stevioside treatment (**Figure 5C**). For MAPK signaling pathways, three major subfamilies of MAPK pathways (ERK, JNK, and p38) were maximally phosphorylated within 30 min of RANKL stimulation, while pretreatment with stevioside significantly attenuated RANKL-induced phosphorylation of these pathways (**Figure 5C**). Quantitative analysis confirmed these observations (**Figure 5D**).

c-Fos and NFATc1 are considered the master long-term signaling regulators of osteoclastogenesis (Gohda et al., 2005). Our results showed that the mRNA and protein levels of c-Fos and NFATc1 were elevated by RANKL stimulation in a time-dependent manner. However, administration of stevioside strongly suppressed the levels of c-Fos and NFATc1 in a time-dependent and dose-dependent manner (**Figures 5E–G**). These data, taken together, demonstrated that stevioside inhibits RANKL-induced osteoclastogenesis by suppressing TAK1 phosphorylation, thus mediating the downstream signaling pathways involved in osteoclastogenesis (**Figure 5H**).

### Stevioside Suppresses Ti Particle-Induced Inflammatory Response *in vitro*

**FIGURE 6:**
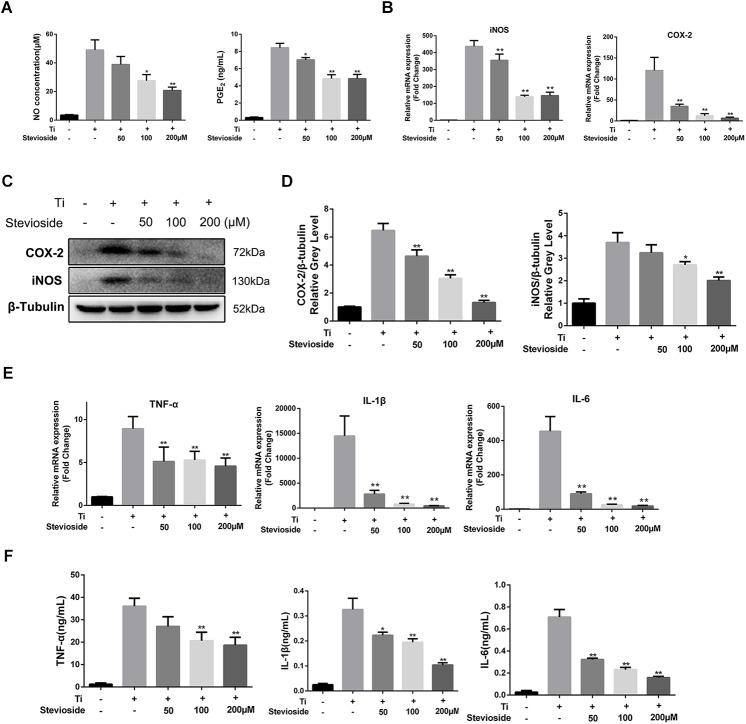
Stevioside inhibits Ti particle-induced inflammatory response *in vitro*. **(A)** BMMs were incubated with Ti particles and the indicated concentrations of stevioside for 24 h. Supernatant was collected, and the production of NO and PGE2 was measured. **(B)** BMMs were incubated with Ti particles and the indicated concentrations of stevioside for 6 h. The mRNA expression of iNOS and COX-2 was measured. **(C)** BMMs were incubated with Ti particles and the indicated concentrations of stevioside for 24 h. The protein levels of iNOS and COX-2 was explored using western blotting. **(D)** The gray levels of iNOS and COX-2 were normalized relative to β-tubulin using ImageJ. **(E,F)** BMMs were incubated with Ti particles and the indicated concentrations of stevioside for 6 or 24 h. The mRNA and protein levels of TNF-α, IL-1β, and IL-6 in BMMs were measured at 6 h for mRNA and 24 h for protein. Data are presented as mean ± SD. ^∗^*P* < 0.05, ^∗∗^*P* < 0.01, compared with Ti particles alone.

To explore the effects of stevioside on Ti particle-induced inflammatory response, we investigated the expression of inflammatory mediators (NO and PGE2) and proinflammatory cytokines (TNF-α, IL-1β, and IL-6) in Ti particle-induced BMMs. As shown in **Figure 6A**, the production of NO and PGE2 was increased by Ti particles stimulation, while stevioside successfully inhibited their production in a dose-dependent manner. Previous studies have demonstrated that iNOS and COX-2 are the key enzymes inducing the production of NO and PGE2 (Caughey et al., 2001; Chauhan et al., 2003). Hence, we further analyzed the expression of COX-2 and iNOS. Our results showed that Ti particles potently elevated the mRNA and protein expression levels of COX-2 and iNOS, and these effects were markedly attenuated by stevioside treatment in a dose-dependent manner (**Figures 6B–D**).

Meanwhile, the effects of stevioside on the release of Ti particle-induced pro-inflammatory cytokines, such as TNF-α, IL-1β, and IL-6, were explored by RT-PCR and ELISA. The mRNA and protein expression of TNF-α, IL-6, and IL-1β were induced by Ti particles and markedly suppressed by stevioside in a concentration-dependent manner (**Figures 6E,F**). Collectively, our data demonstrated that stevioside exhibited great effects on suppressing inflammatory response in Ti particle-induced BMMs, which was consistent with the *in vivo* results.

### Stevioside Suppresses Ti Particle-Induced NF-κB and MAPK Signaling Pathways Through the Inhibition of TAK1 Phosphorylation

**FIGURE 7:**
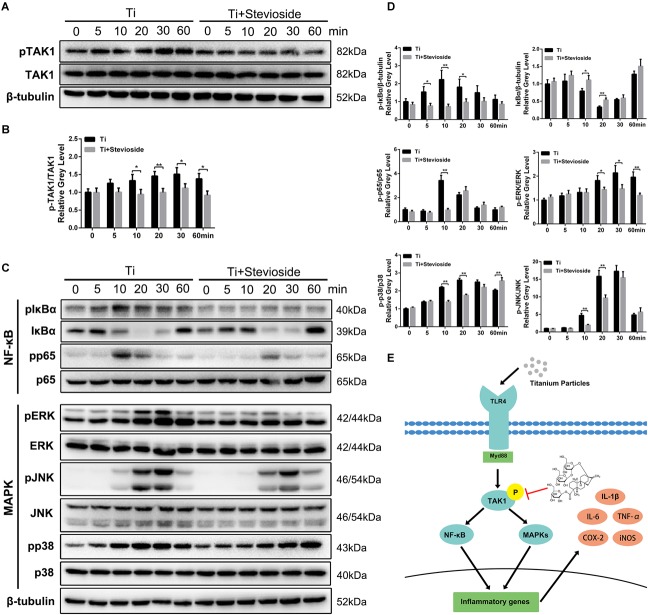
**(A,C)** BMMs were pretreated with or without 200 μM stevioside for 4 h and then treated with 0.1 mg⋅mL^-1^ Ti particles for the indicated periods. Cell lysates were analyzed using western blotting. **(B)** The gray level of phosphorylated TAK1 was normalized relative to TAK1. **(D)** The gray levels of phosphorylated p65, ERK, JNK, and p38 were quantified and normalized relative to total p65, ERK, JNK, and p38. The gray levels of phosphorylated IκBα and IκBα were normalized to β-tubulin. **(E)** Schematic representation of the experiments presented in this figure. Data are presented as mean ± SD. ^∗^*P* < 0.05, ^∗∗^*P* < 0.01, compared with Ti particles alone.

Previous studies have demonstrated that the activation of TAK1 and subsequent signaling cascades (NF-κB and MAPK pathways) play a crucial role in Ti particle-induced inflammatory response in macrophages, and targeted suppression of TAK1 strongly impairs these pathways, thereby reducing the production of pro-inflammatory cytokines (Cheng et al., 2010; Landgraeber et al., 2014). Due to the remarkable suppressive effects of stevioside on RANKL-induced TAK1 phosphorylation, we hypothesized that stevioside could also inhibit Ti particle-induced phosphorylation of TAK1 and subsequent signaling cascades. As expected, the phosphorylation of TAK1 was observed 5 min after Ti particles stimulation and peaked at 30 min. In contrast, the administration of stevioside effectively inhibited the phosphorylation of TAK1 induced by Ti particles (**Figure 7A**), and quantitative analysis confirmed our observation (**Figure 7B**).

In addition, the signaling cascades following TAK1 activation were also detected. As shown in **Figure 7C**, both NF-κB (IκBα and p65) and MAPK (ERK, JNK, and p38) were phosphorylated by the stimulation of Ti particles, while the phosphorylation levels were significantly suppressed by stevioside treatment. Quantitative analysis also confirmed these observations (**Figure 7D**). To summarize our results, stevioside had a remarkable effect on inhibiting Ti particle-induced NF-κB and MAPK pathways by targeting TAK1 phosphorylation (**Figure 7E**).

## Discussion

TJA is widely utilized for the treatment of end-stage joint disease. However, arthroplasty failure increases with time because of wear particle-induced periprosthetic osteolysis and subsequent aseptic loosening (Bozic et al., 2009; Beck et al., 2012). Although bisphosphonates and teriparatide have been reported to inhibit wear particle-induced osteolysis in animal models, the side effects, such as fever, gastrointestinal toxicity, osteonecrosis of the jaw, osteosarcoma et al. and high cost limit their clinical application (Millett et al., 2002; Wedemeyer et al., 2005; Bi et al., 2015). Here we report for the first time that stevioside, a natural component from *Stevia rebaudiana*, protected against wear particle-induced osteolysis.

Wear particle-induced osteolysis is a complex pathophysiological process that involves various cell types and inflammatory cytokines. Generally, increased inflammatory response and elevated numbers of osteoclasts may be responsible for the periprosthetic bone loss (Greenfield et al., 2002; Hirayama et al., 2002; Helfrich, 2005). In this study, we developed Ti particle-induced osteolysis in a mouse calvarial model. The presence of Ti particles induced severe erosion of calvarial bone, as indicated by extensive bone resorption observed using micro-CT and 3D reconstruction. In contrast, stevioside-treated mice showed dose-dependent suppression of particle-induced osteolysis. Histological analysis of sections stained with H&E and TRAP showed that stevioside inhibited Ti particle-induced bone erosion and osteoclast formation. In addition, we also demonstrated that stevioside inhibited Ti particle-induced inflammatory response by evaluating the mRNA and protein levels of inflammatory genes in parietal bones. The suppressive effects of stevioside on osteoclastogenesis and inflammatory response were also confirmed *in vitro*. Our *in vitro* results showed that stevioside inhibited the differentiation of BMMs into mature osteoclasts and attenuated the bone resorption of mature osteoclasts. Moreover, the proinflammatory cytokines and inflammatory mediators increased by Ti particles were significantly suppressed by stevioside treatment. Mechanistically, stevioside inhibited the phosphorylation of TAK1 and subsequent activation of NF-κB/MAPK signaling pathways in RANKL- or Ti-induced signaling cascades in BMMs. Taken together, our findings suggest that stevioside may be a novel drug applied for the prevention or treatment of periprosthetic osteolysis and other osteolytic diseases.

In the osteoclast differentiation of mammalian cells, the activation of TAK1 is an important signaling event in RANKL signaling pathway (Huang et al., 2006; Sumiya et al., 2015). Generally, binding of RANKL to its receptor RANK promotes the recruitment of tumor necrosis factor receptor-associated factor 6 (TRAF6) and then forms a signaling complex containing RANK and TAK1-binding protein (TAB)2, resulting in TAK1 phosphorylation. Activated TAK1 is able to phosphorylate both IκB kinase (IKK) and MAPK kinases (MKKs) to initiate NF-κB and MAPK signaling pathways, which are important and indispensable for osteoclastogenesis (Gohda et al., 2005; Idris et al., 2010; Stevenson et al., 2011). In our study, we observed that stevioside suppressed the NF-κB signaling pathway induced by RANKL through the inhibition of the phosphorylation of IκBα and p65 as well as the proteasomal degradation of IκBα. In addition, stevioside inhibited the phosphorylation of all three MAPK pathways (ERK, JNK, and p38) in BMMs stimulated with RANKL. Li et al. reported that stevioside inhibited the release of LPS-induced pro-inflammatory cytokines by interfering with both NF-κB and MAPK signaling pathways (Fengyang et al., 2012). Although there are considerable differences between LPS and RANKL in their receptors and intracellular signaling pathways, the activation of TAK1 plays a crucial role in both LPS-induced and RANKL-induced signaling cascades (Irie et al., 2000; Huang et al., 2006). Based on our observations and previous research by Li et al., we further investigated the effect of stevioside on TAK1 phosphorylation. As expected, stevioside significantly suppressed the phosphorylation of TAK1, which was a probable reason for its inhibition effects on NF-κB and MAPK signaling pathways.

Ti particle is a common type of wear debris in TJA that induces macrophages to produce inflammatory mediators and inflammatory cytokines such as TNF-α, IL-1β, IL-6, NO, and PGE2 (Suzuki et al., 2007; Bechtel et al., 2016). Previous studies have reported that TLR4/Myd88 plays an important role in titanium particle-induced inflammation and osteolysis (Takagi et al., 2007; Obando-Pereda et al., 2014). A previous study demonstrated that Ti particles can activate TAK1-NF-κB/MAPK signaling pathway in RAW264.7 cells, and targeted suppression of TAK1 strongly impairs these pathways, thereby reducing the production of pro-inflammatory cytokines (Cheng et al., 2010). Our results showed that stevioside suppressed the phosphorylation of TAK1 in Ti particle-induced BMMs as well as the subsequent activation of NF-κB and MAPK pathways. In addition, stevioside significantly inhibited the secretion of TNF-α, IL-6, IL-1β, NO, and PGE2 and the intracellular expression of iNOS and COX2 in a dose-dependent manner, suggesting that stevioside could prevent Ti particle-induced osteolysis by inhibiting localized release of inflammatory mediators and inflammatory cytokines.

Nevertheless, there are several limitations of this study. First, UHMWPE wear particles are a more common cause of TJA failure than metal particles (Hirakawa et al., 1996). However, metal particles are still an important factor contributing to osteolysis, and both metal and UHMWPE particles could induce osteolysis *in vivo* (Wooley et al., 2002; von Knoch et al., 2004). Therefore, it is reasonable to establish the osteolytic animal model using Ti particles, even though UHMWPE particles would be more closely related to the clinical findings. Second, wear particle-induced osteolysis *in vivo* is a complex process involving many types of cells, especially macrophages, osteoclasts and osteoblasts (Purdue et al., 2007). Here, we investigated the effects and mechanisms of stevioside on macrophages and osteoclasts, and further studies are needed to address its effect on osteoblasts.

In conclusion, our results showed that stevioside inhibits inflammatory response and osteoclastogenesis both *in vitro* and *in vivo*. The suppressive effects are achieved through the inhibition of TAK1 phosphorylation and subsequent activation of NF-κB/MAPK signaling pathways. Therefore, our data suggest that stevioside is a potential drug for the prevention or treatment of periprosthetic osteolysis and other osteolytic diseases.

## Materials and Methods

### Media and Reagents

Stevioside (purity >98%, **Supplementary Figure S1**) was purchased from Sigma-Aldrich (St. Louis, MO, United States). Alpha modification of Eagle’s medium (α-MEM), Dulbecco’s modified Eagle’s medium (DMEM), fetal bovine serum (FBS), and penicillin/streptomycin were purchased from Gibco-BRL (Gaithersburg, MD, United States). The cell counting kit-8 (CCK-8) was obtained from Dojindo Molecular Technology (Kumamoto, Japan). Recombinant mouse macrophage colony-stimulating factor (M-CSF) and RANKL were obtained from R&D Systems (Minneapolis, MN, United States). Specific antibodies against p38 (#9212), phospho-p38 (Thr180/Tyr182) (#4511), ERK (#4695), phospho-ERK (Thr202/Tyr204) (#4370), JNK 1/2 (#9252), phospho-JNK (Thr183/Tyr185) (#4668), IκBα (#4814), phospho-IκBα (Ser32) (#2859), p65 (#8242), phospho-p65 (Ser536) (#3033), TAK-1 (#5206), phosphop-TAK1 (Thr184/187) (#4508), c-Fos (#2250), nuclear factor of activated T cells c1 (NFATc1) (#8032), and β-tubulin (#2146) were obtained from Cell Signaling Technology (Danvers, MA, United States). Specific antibodies against inducible nitric oxide synthase (iNOS) (sc-7271) and cyclooxygenase-2 (COX-2) (sc-166475) were obtained from Santa Cruz Biotechnology (Santa Cruz, CA, United States). Commercial enzyme-linked immunosorbent-based assay (ELISA) kits for TNF-α, IL-1β, and IL-6 detection were obtained from R&D Systems. TRAP staining kit, and other reagents were purchased from Sigma-Aldrich unless otherwise noted.

### Ti Particle-Induced Murine Calvarial Osteolysis Model

All experimental procedures were performed in accordance with the principles and procedures of the National Institutes of Health (NIH) Guide for the Care and Use of Laboratory Animals, and the Guide of the Animal Care Committee of Zhejiang University. Sixty healthy 6-week-old male C57BL/6 mice weighing 18–22 g were obtained from the Experimental Animal Center of Zhejiang University. All animals were housed in a room at 22 ± 2°C, 60% humidity and 12: 12 h light-dark cycle with free access to food and water, with five animals per cage.

A Ti particle-induced calvarial osteolysis model was established to determine the effects of stevioside on osteolysis *in vivo*. This mouse model has been in use for studying the pharmacological effects of drugs on particle-induced osteolysis for some years (Zhang et al., 2001; Tian et al., 2014; Wu et al., 2015) and is not replaceable with *in vitro* culture systems. Commercially available pure Ti particles were obtained from Johnson Matthey (Ward Hill, MA, United States). Particles were prepared by baking at 180°C and subsequent mixed with 70% ethanol for 48 h to remove endotoxin and ensure sterility (Zhou et al., 2018). After acclimatizing to the laboratory for 1 week, mice were randomly divided into four experimental groups (*n* = 15 per group): PBS control (sham), Ti particles in PBS(vehicle), Ti particles together with low (low dose, 10 mg⋅kg^-1^⋅day^-1^) and high (high dose, 30 mg⋅kg^-1^⋅day^-1^) doses of stevioside. The doses of stevioside were determined according to previous research (Yingkun et al., 2013; Ragone et al., 2017). After mice were anesthetized with intraperitoneal sodium pentobarbital (50 mg⋅kg^-1^), the cranial periosteum was separated, and 30 mg of Ti particles in PBS were embedded under the periosteum at the middle suture of the calvaria in the vehicle group and stevioside groups, while PBS was injected in the sham group. One day after implantation, PBS or stevioside was administrated intragastrically every day for 2 weeks. At the end of the experiment, the mice were sacrificed with an overdose of sodium pentobarbital (120 mg⋅kg^-1^), and the calvaria were harvested for subsequent analysis.

### Micro-CT Scanning

The calvaria were analyzed (*n* = 5 per group) using a high-resolution micro-CT (Skyscan 1072, Aartselaar, Belgium). The scanning protocol was set at an isometric resolution of 9 μm and X-ray energy settings of 80 kV and 80 μA. Three-dimensional (3D) reconstruction was then performed, and a square region of interest (ROI, 3 mm × 3 mm) around the midline suture was selected for further qualitative and quantitative analysis. BV/TV, number of porosity, and percentage of porosity for each specimen were measured in the ROI as reported previously (Wedemeyer et al., 2007).

### H&E and TRAP Staining of Tissue Sections

Calvarial samples (*n* = 5 per group) were decalcified in 10% EDTA (pH = 7.4) for 2 weeks and then embedded in paraffin. Histological sections were prepared for TRAP and H&E staining. The specimens were examined and photographed under a Nikon Eclipse TE2000-S microscope (Tokyo, Japan). BV/TV, erosion area, the number of TRAP-positive osteoclasts, and OcS/BS were assessed for each sample.

### Calvaria Culture

As previously described (Zhai Z. et al., 2014; Shao et al., 2015), calvaria (*n* = 5 per group) were harvested after the implantation with Ti particles for 2 weeks, under sterile conditions, and each was placed into one well of a 6-well plate containing 2 mL of DMEM with 100 U⋅mL^-1^ penicillin and 100 mg⋅mL^-1^ streptomycin. Calvaria were incubated at 37°C with 5% CO_2_ for 24 h, and then the culture medium was collected and stored at -80°C for ELISA.

### Cell Culture

Monocyte/macrophage precursors were obtained from femur and tibia bone marrow of 6-week-old male C57BL/6 mice as described previously (Wang et al., 2014; Zhai Z.J. et al., 2014) and then differentiated into BMMs in complete α-MEM (10% FBS, 100 U⋅mL^-1^ penicillin and 100 μg⋅mL^-1^ streptomycin) supplemented with 25 ng⋅mL^-1^ M-CSF for 5 days. All cell cultures were maintained in a humidified environment of 5% CO_2_ at 37°C.

### Cell Viability Assay

The effects of stevioside on BMMs viabilities were determined using a CCK-8 assay. Cells were seeded in a 96-well plate at a density of 5 × 10^3^ cells per well and cultured in complete α-MEM medium for 48 h or 96 h in the presence of increasing concentrations of stevioside (0–400 μM). Next, 10 μL CCK-8 buffer was added to each well, and the plate was incubated for another 2 h. The optical density (OD) was measured at a wavelength of 450 nm (650 nm reference) with an ELX800 absorbance microplate reader (Bio-Tek, Winooski, VT, United States). The viabilities of BMMs exposed to stevioside were expressed as a percentage of untreated cells.

### *In vitro* Osteoclast Differentiation

BMMs were seeded into a 96-well plate at a density of 8 × 10^3^ cells per well, in complete α-MEM supplemented with 25 ng⋅mL^-1^ M-CSF, 50 ng⋅mL^-1^ RANKL, and different concentrations of stevioside (0, 50, 100, and 200 μM). Culture medium was replaced every 2 days. After 6 days of culture, cells were washed twice with PBS, fixed with 4% paraformaldehyde (PFA), and stained for TRAP. TRAP-positive multinucleated cells with more than five nuclei were counted under a light microscope.

### F-Actin Ring Immunofluorescence and Resorption Pit Assay

To visualize F-actin rings, BMMs were treated with 25 ng⋅mL^-1^ M-CSF and 50 ng⋅mL^-1^ RANKL for 4 days. An equal number of BMM-derived osteoclasts were seeded onto bovine bone slices and allowed to adhere overnight. Cells were then treated with different concentrations of stevioside (0, 50, 100, and 200 μM) for another 2 days. Next, cells were fixed with 4% PFA for 15 min, permeabilized for 5 min with 0.5% Triton X-100, and stained with rhodamine-conjugated phalloidin (1:200; Invitrogen Life Technologies, Carlsbad, CA, United States) diluted in 0.2% bovine serum albumin (BSA)–PBS for 1 h. Fluorescent images were captured with a fluorescence microscope (EU5888, Leica, Wetzlar, Germany) and analyzed using ImageJ software (National Institutes of Health, Bethesda, MD, United States). Then, these bone slices were washed twice with PBS for the resorption pit assay. Cells that had adhered to the bone slices were removed by mechanical agitation. Bone slice images were captured using a scanning electron microscope (SEM; S-4800, Hitachi, Japan) and analyzed using ImageJ software.

### Particle-Induced Inflammatory Response *in vitro*

Ti particles were used to induce the inflammatory response in BMMs. The time points used for our experiments were based on previous research (Bechtel et al., 2016). BMMs were seeded into a 6-well plate at a density of 5 × 10^5^ cells per well, in complete α-MEM supplemented with 25 ng⋅mL^-1^ M-CSF and allowed to adhere overnight. Then, Ti particles (0.1 mg⋅mL^-1^) and different concentrations of stevioside (0, 50, 100, and 200 μM) were added into the culture medium. After incubation for 6 h, total RNA from the adherent cells was extracted for RT-PCR analysis. After incubation for 24 h, the supernatants were collected for ELISA analysis, and the protein from adhered cells was extracted for western blotting.

### RNA Extraction and Quantitative PCR Assay

Total RNA from calvaria (*n* = 5 per group) or cultured cells was extracted using the Qiagen RNeasy Mini Kit (Qiagen, Valencia, CA, United States) following the manufacturer’s protocols. Complementary DNA (cDNA) was synthesized from 1 μg of total RNA using reverse transcriptase (TaKaRa Biotechnology, Otsu, Japan). Real-time PCR was performed using the SYBR Premix Ex Tag Kit (TaKaRa Biotechnology) and the ABI 7500 Sequencing Detection System (Applied Biosystems, Foster City, CA, United States). Each reaction was run for 40 cycles at the following conditions: 95°C for 5 s, 60°C for 20 s, and 72°C for 20 s. *GAPDH* was used as a housekeeping gene. The mouse primer sequences are shown in **Table 1**.

**Table 1:** Primers used for quantitative real-time PCR.

Gene	Forward (F) and reverse (R) primer sequence (5′–3′)
GAPDH	F: ACCCAGAAGACTGTGGATGG
	R: CACATTGGGGGTAGGAACAC
CTSK	F: CTTCCAATACGTGCAGCAGA
	R: TCTTCAGGGCTTTCTCGTTC
TRAP	F: CTGGAGTGCACGATGCCAGCGACA
	R: TCCGTGCTCGGCGATGGACCAGA
DC-STAMP	F: AAAACCCTTGGGCTGTTCTT
	R: AATCATGGACGACTCCTTGG
c-Fos	F: CCAGTCAAGAGCATCAGCAA
	R: AAGTAGTGCAGCCCGGAGTA
NFATc1	F: CCGTTGCTTCCAGAAAATAACA
	R: TGTGGGATGTGAACTCGGAA
TNF-α	F: CATCTTCTCAAAATTCGAGTGACA
IL-1β	R: TGGGAGTAGACAAGGTACAACCC
IL-6	F: TGCCACCTTTTGACAGTGATG
iNOS	R: TGATGTGCTGCTGCGAGATT
COX-2	F: TCCAGTTGCCTTCTTGGGAC
	R: AGTCTCCTCTCCGGACTTGT
	F: CATGCTACTGGAGGTGGGTG
	R: CATTGATCTCCGTGACAGCC
	F: CCCGGACTGGATTCTATG
	R: AACCCAGGTCCTCGCTTATGA

### Western Blotting

Western blotting was used to determine the main signaling pathways affected by stevioside. BMMs were seeded in a 6-well plate at a density of 8 × 10^5^ cells per well. After pretreatment with or without 200 μM stevioside for 4 h, cells were stimulated with 50 ng⋅mL^-1^ RANKL or 0.1 mg⋅mL^-1^ Ti particles for 0, 5, 10, 20, 30, or 60 min. To examine the effects of stevioside on c-Fos and NFATc1 expression, BMMs were plated in a 6-well plate at a density of 1 × 10^5^ cells per well and cultured with 25 ng⋅mL^-1^ M-CSF and 50 ng⋅mL^-1^ RANKL in the presence or absence of 200 μM stevioside for 0, 2, 4, or 6 days. To investigate the effects of stevioside on Ti particle-induced expression of iNOS and COX-2, BMMs were cultured in 6-well plates at a density of 8 × 10^5^ cells per well in complete α-MEM containing 25 ng⋅mL^-1^ M-CSF and different doses of stevioside (0, 50, 100, and 200 μM) with or without 0.1 mg⋅mL^-1^ Ti particles for 24 h. Total protein was extracted from cultured cells using radioimmunoprecipitation assay (RIPA) lysis buffer (Sigma-Aldrich). Each protein lysate containing 30 μg protein was separated using 10% sodium dodecyl sulfate-polyacrylamide gel electrophoresis (SDS-PAGE) and transferred to polyvinylidene difluoride membranes (Millipore, Bedford, MA, United States). After non-specific blocking for 1 h, membranes were incubated with primary antibodies at 4°C overnight. After three washes with TBS-Tween, we subsequently incubated membranes with the appropriate secondary antibodies at 4°C for 2 h. The signals were detected by exposure in a Bio-Rad XRS chemiluminescence detection system (Hercules, CA, United States).

### Enzyme-Linked Immunosorbent Assay

ELISA was conducted to detect the relative cytokine levels in cultured calvaria or cultured cells. The culture medium was collected, centrifuged (2300 ×*g*, 25 min), passed through a 0.2 μm filter (Millipore), and stored at -80°C until use. Mouse ELISA kits were used to determine TNF-α, IL-1β, and IL-6 concentrations in accordance with the manufacturer’s instructions. Absorbance was measured using an ELX800 absorbance microplate reader at 450 nm. The detection limits of the assay were 7.21 pg⋅mL^-1^ for TNF-α, 4.8 pg⋅mL^-1^ for IL-1β, 1.8 pg⋅mL^-1^ for IL-6.

### Statistical Analysis

All data are expressed as mean ± standard deviation (SD). Each experiment was repeated at least three times separately and the results were analyzed with Prism 6.01 (GraphPad Software, La Jolla, CA, United States). A two-tailed, unpaired Student’s *t*-test was used for the comparisons between two groups. One-way ANOVA with *post hoc* Newman-Keuls test was used to analyze differences in multiple comparisons. Values of *P* < 0.05 were considered statistically significantly different.

## Author Contributions

JM, SY, and WY designed the research. JM, CZ, WW, GJ, and JH performed *in vitro* research. JM, BH, YY, and YW performed the animal study. HW and SL analyzed the data. JM and ML wrote the paper. Experiments were performed under the supervision of HW, SY, and WY.

## Conflict of Interest Statement

The authors declare that the research was conducted in the absence of any commercial or financial relationships that could be construed as a potential conflict of interest.

## Supplementary Material

FIGURE S1 The structure of stevioside.
