# Supplementary figures and images for: Stevioside Prevents Wear Particle-Induced Osteolysis by Inhibiting Osteoclastogenesis and Inflammatory Response via the Suppression of TAK1 Activation
Source: Front Pharmacol. 2018 Sep 26;9:1053. doi: 10.3389/fphar.2018.01053 (PMC6169369; doi:10.3389/fphar.2018.01053)

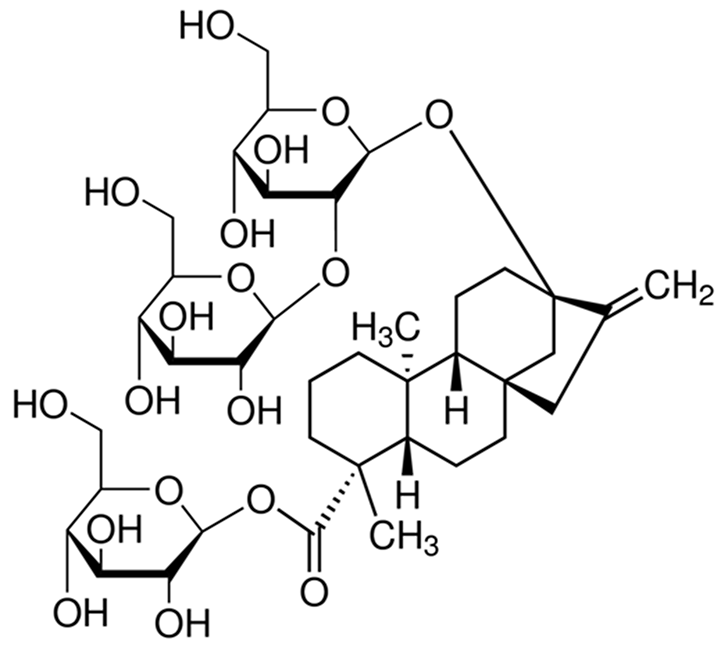

Supplement: FIGURE S1 — The structure of stevioside. [file Image_1.TIF]
